# Transforming Paper into Plasmonic Sensors: One‐Step Fabrication of High‐Enhancement SERS Nanosubstrates via Surface Energy Control

**DOI:** 10.1002/smtd.70803

**Published:** 2026-06-26

**Authors:** Farbod Ebrahimi, Anjali Kumari, Kyle Nowlin, Tohid Didar, Kristen Dellinger

**Affiliations:** ^1^ Department of Nanoengineering Joint School of Nanoscience and Nanoengineering North Carolina A&T State University Greensboro North Carolina USA; ^2^ Department of Nanoscience Joint School of Nanoscience and Nanoengineering University of North Carolina at Greensboro Greensboro North Carolina USA; ^3^ Department of Mechanical Engineering McMaster University Hamilton Ontario Canada

**Keywords:** cellulose‐based biosensor, hydrophobicity, nanofabrication, physical vapor deposition, Raman spectroscopy, rhodamine B, silver nanoparticle

## Abstract

Surface‐enhanced Raman scattering (SERS) substrates require precise control of plasmonic nanoparticles to generate hot spots for ultrasensitive detection. We present the Surface‐Induced Layered Vapor Energy Refined Enhanced Deposition (SILVERED) method for fabricating silver nanoparticle‐decorated paper substrates through single‐step physical vapor deposition on fluorosilanized cellulose. The extreme surface energy differential drives Volmer‐Weber growth, yielding monodisperse (29 ± 4 nm) nanoparticles with high uniformity (PDI = 0.13). Optimal deposition achieved 10^6^ enhancement factor and 2 pM detection limit for rhodamine B. The hydrophobic surface enabled analyte pre‐concentration while maintaining reproducibility (RSD < 10%), and the analytical metrics include contributions from substrates drawn across independent fabrication batches. Substrates retained more than 95% of their performance under nitrogen storage. The method's scalability (12‐inch substrates), reproducibility, hydrophobicity, silver nanoparticle size, and low cost position it for diverse applications beyond SERS.

## Graphical abstract

Cellulose paper is transformed into a high‐performance plasmonic sensor through a process called SILVERED, which involves single‐step vapor deposition onto fluorosilanized fibers. Surface‐energy contrast drives uniform silver nanoparticle growth with sub‐10 nm hot‐spot gaps, achieving 2 pM detection of rhodamine B. Hydrophobicity self‐focuses analytes to deliver a sensor platform with potential applications for diagnostics, food safety, and environmental monitoring.

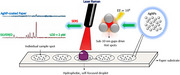

## 1 Introduction

Cellulose paper is a renewable, biodegradable, and globally abundant material that has been advocated as a sustainable foundation for next‐generation sensors and flexible electronics, offering a credible response to the rapid growth of electronic waste from conventional rigid platforms [1]. Silver nanoparticle (AgNP)‐decorated cellulose substrates have emerged as versatile platforms for applications ranging from catalysis to sensing [2, 3, 4]. While the fibrous architecture of paper provides high surface area and interconnected porosity, AgNPs contribute plasmonic activity and antimicrobial functionality [2, 5]. Despite this potential, achieving uniform NP distribution with controlled morphology through reproducible and scalable methods remains challenging.

The controlled fabrication of metallic NP arrays on 3D substrates requires precise manipulation of nucleation and growth processes. 3D fibrous substrates offer benefits such as a larger active volume and better electromagnetic field confinement, but achieving uniform NP formation on these complex architectures remains technically demanding. This is particularly true for cellulose‐based materials, where the inherently hydrophilic nature leads to uncontrolled wetting during conventional deposition. The hydrophilic nature of filter paper causes rapid absorption and dispersion of NPs throughout the matrix [6].

The need for ultrasensitive detection extends across multiple domains. In clinical settings, disease biomarkers typically exist at extremely low concentrations [3, 4]. Similarly, detecting contaminants such as rhodamine B (RhB) in food requires ultrasensitive methods as it remains colorless, even at harmful concentrations [7, 8]. Current detection methods rely on complex laboratory techniques demanding specialized facilities and high costs [9, 10]. Surface‐enhanced Raman scattering (SERS) combines molecular specificity, minimal sample preparation, and rapid analysis, positioning it as an ideal analytical platform [4].

Current fabrication methods face significant limitations in producing uniform AgNP‐coated paper at scale, especially for SERS applications. Although SERS offers remarkable detection capabilities [4, 10], developing substrates with uniform hot spots remains challenging. Traditional 2D substrates suffer from limited reproducibility due to the confined confocal laser volume [11]. As a result, 3D SERS substrates have emerged as superior alternatives, offering increased hot spot density and improved signal enhancement [12, 13, 14]. Despite remarkable fundamental advancements in SERS over the past 50 years, translation to commercial products has been surprisingly slow due to persistent challenges in substrate stability, reproducibility, and manufacturing cost [15].

Various fabrication methods, including self‐assembly [16], immersion and chemical reduction [17, 18], electrostatic adsorption [19], filtration [20], NP aggregation, vapor deposition [21], inkjet printing [22], and laser annealing [23] have been developed, but each has limitations. Chemical methods struggle with uniformity, while physical methods face challenges with temperature sensitivity [24]. Recent laser‐based fabrication shows promise [23], but high equipment costs and limited processing areas present barriers to widespread commercial adoption. Within paper‐based SERS, two strategies dominate the recent literature. Oliveira et al. drop‐cast Ag nanostars onto hydrophilic‐patterned office paper to reach an enhancement factor (EF) of 10^7^ for rhodamine 6G (R6G), but require wet‐chemical nanostar synthesis and hydrophilic patterning [25]. Ozório et al. coupled hydrothermally grown ZnO nanorods on Whatman paper with dewetted AgNPs to reach EFs approaching 10^7^ for environmental contaminants, through two sequential nanostructure‐formation steps [26]. Both deliver strong sensitivity but rely on multi‐step wet chemistry that constrains reproducibility and scalability.

**FIGURE 1:**
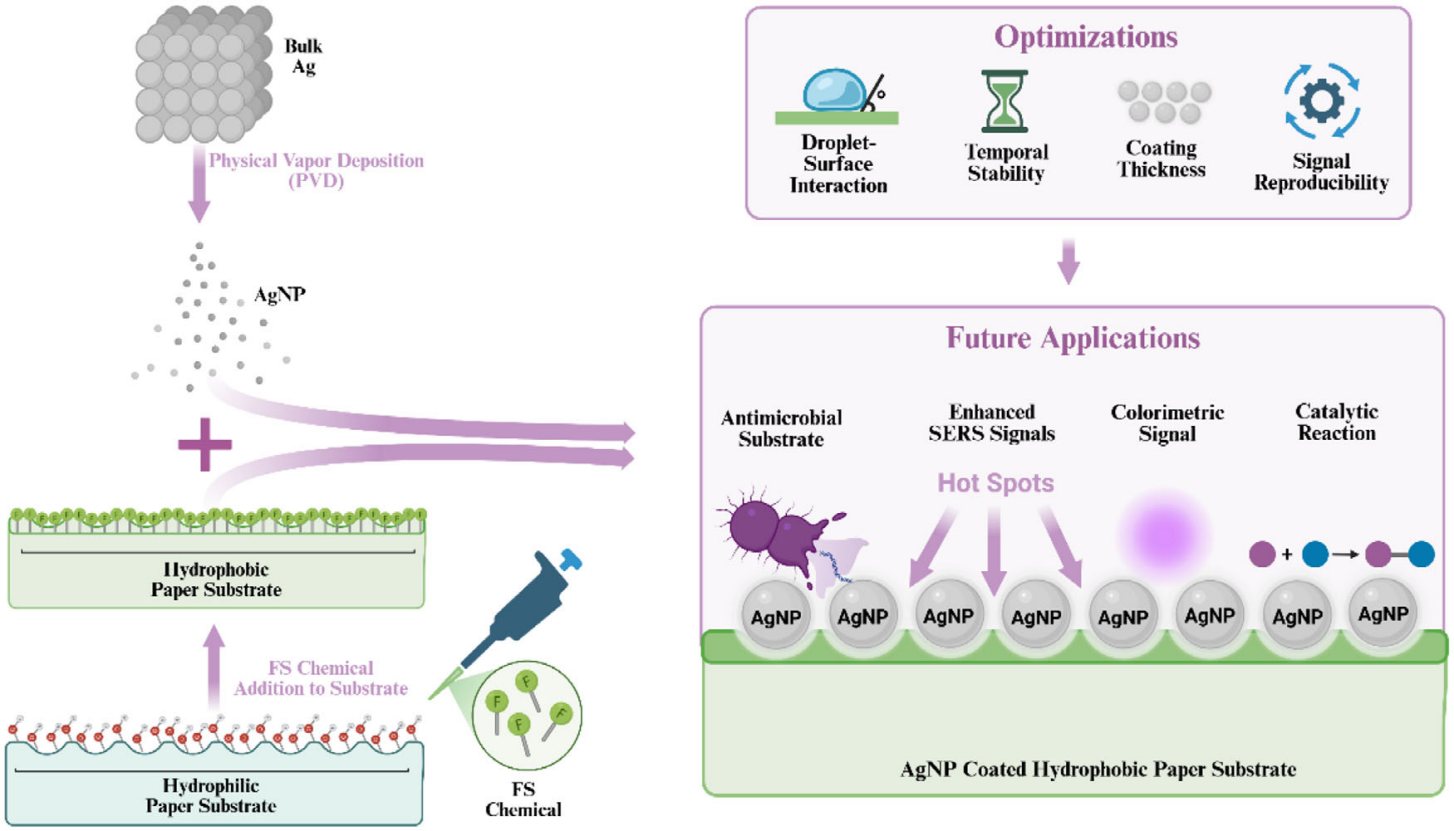
Schematic illustration of the SILVERED method showing the transformation from hydrophilic paper to hydrophobic fluorosilanized substrate, followed by AgNPs deposition via PVD (created by Biorender.com).

Here, we introduce the Surface‐Induced Layered Vapor Energy Refined Enhanced Deposition (SILVERED) method (Figure 1), a top‐down, single‐step dry process that sets the AgNP size, density, and inter‐particle distance thermodynamically through the surface‐energy contrast between fluorosilanized cellulose and silver, with no colloid synthesis, no patterning, no metal‐oxide scaffolding, and no post‐deposition treatment. The method scales to 12 inches, overcoming typical wafer‐scale constraints [24]. A single 12‐inch substrate yields approximately 2800 individual sensors (5 × 5 mm) at less than ∼$0.10 per test, representing a significant cost advantage over conventional fabrication methods. The key innovation lies in fluorosilane treatment of cellulose fibers, enabling direct AgNP formation without additional processing. The significant surface energy differential induces thermodynamically driven formation of discrete NPs rather than continuous films [27]. The retained hydrophobicity creates a concentration effect that focuses analytes during evaporation, improving sensitivity [28]. By utilizing inexpensive filter paper as the substrate material and eliminating post‐deposition treatments, we significantly reduce manufacturing complexity and costs compared to conventional approaches. The effectiveness of our SILVERED approach is demonstrated through the ultrasensitive detection of RhB, showcasing the substrate's capability for detecting food contaminants at concentrations relevant for safety monitoring. The 3D paper‐based SERS substrates fabricated through the straightforward SILVERED approach hold significant potential as rapid, cost‐effective, label‐free, and highly sensitive detection tools for diverse applications from chemical analysis to environmental monitoring and integration into point‐of‐care devices.

## 2 Results and Discussion

### 2.1 Surface Energy‐Controlled Nanoparticle Formation

The SILVERED approach uses single‐step physical vapor deposition(PVD) on modified cellulose paper. Surface modification using fluorosilane transforms the hydrophilic paper by replacing hydroxyl groups with fluorinated moieties, reducing surface energy [24]. Three fabrication parameters control SILVERED performance: the completeness of fluorosilanization, which sets the surface‐energy contrast that drives Volmer–Weber growth; the nominal Ag thickness, which sets the equilibrium NP size and density; and the PVD deposition rate, which governs the kinetic competition between nucleation of new islands and growth of existing ones. The silanization protocol and the deposition rate (0.1 Å/s) were held fixed, and the nominal thickness was used as the single tunable parameter; the resulting morphological progression directly determines the analytical metrics discussed in Section 2.3. The substantial energy difference between the modified substrate and silver drives controlled NP array formation through Volmer‐Weber growth [24, 29]. Silver atoms preferentially nucleate as discrete NPs during deposition, forming dense arrays with optimally sized nanogaps that serve as electromagnetic hot spots.

**FIGURE 2:**
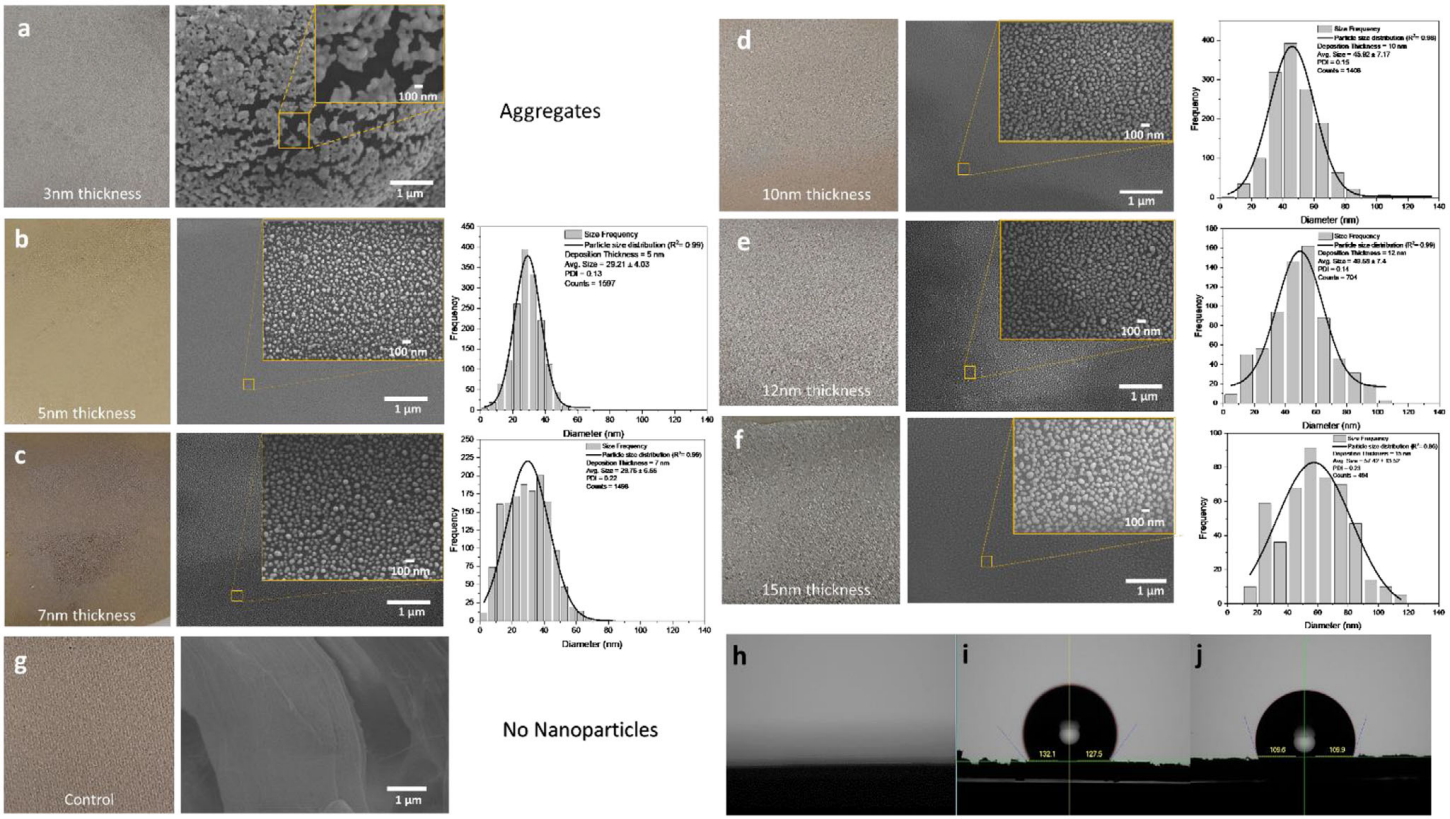
Characterization of AgNP evolution via SILVERED approach at different thicknesses. (a–f) Substrate photographs (left), SEM images (center, scale bars: 1 µm and 100 nm), and corresponding size distribution histograms (right) for: (a) 5 nm, (b) 7 nm, (c) 10 nm, (d) 12 nm, (e) 15 nm Ag deposition thicknesses, and (f) control sample without Ag deposition. (h–j) Water CA measurements: (h) pristine substrate (0°), (i) fluorosilanized surface (127.5°), (j) silver‐coated nanosubstrate (110.4°). Particle size distributions in (a–f) were calculated from *n* > 500 particles per condition using ImageJ; data are reported as mean ± SD.

After fluorosilanization, the contact angle (CA) measurements showed a controlled transformation from complete wetting (0°) to 127.5°, which is close to superhydrophobic behavior (above 150°) (Figure 2h–j). Aqueous droplets are restricted to compact geometries by this extreme hydrophobicity, which stops them from spreading laterally across the substrate surface (Figures S3–S5). During evaporation, analyte molecules are concentrated at the droplet contact line rather than spreading out over wide areas, effectively pre‐concentrating target species at SERS‐active sites. Substrates maintained their 110.4°CA after silver deposition, which is essential for preserving analyte concentration and droplet confinement.

Systematic investigation across 3–15 nm depositions revealed critical structure‐performance relationships. At 3 nm, the deposited silver exhibited island growth rather than uniform NP distribution (Figure 2a). On the hydrophobic surface, insufficient silver atoms initially reached the substrate to establish adequate nucleation sites for individual particle formation. Consequently, subsequently deposited silver preferentially migrated to these limited nucleation sites, forming large aggregated clusters through layer‐by‐layer island growth (Volmer‐Weber mechanism). Additionally, at such ultra‐thin depositions, surface heterogeneities (e.g., localized defects or pores) may preferentially serve as nucleation sites, concentrating silver deposition into discrete islands. It should be noted that the specific island formation behavior at very thin depositions may be influenced by PVD parameters such as deposition rate and chamber conditions, which can affect nucleation kinetics. This resulted in bright, prominent aggregates with significant bare substrate areas between them.

The 5 nm deposition demonstrated optimal uniformity with 29 ± 4 nm NPs and the highest density (Figure 2b). A PDI of 0.13 indicates that 68% of particles fall within ±4 nm of the mean size, ensuring uniform electromagnetic enhancement across the substrate. This uniformity is critical for quantitative SERS, as EFs scale non‐linearly with particle size; small morphological variations can cause order‐of‐magnitude differences in signal intensity between adjacent regions. The 5 nm thickness provides sufficient material to overcome hydrophobic barriers while preventing excessive Ostwald ripening.

At 7 nm, despite similar dimensions (29 ± 5 nm), the system showed 69% increased polydispersity (PDI = 0.22) and reduced density (Figure 2c). With a PDI of 0.22, approximately 32% of NPs deviate significantly from the mean size, creating spatially heterogeneous enhancement. This greater heterogeneity implies that the additional silver content begins to disrupt the delicate balance of controlled nucleation established at 5 nm thickness. The growth mechanism shifts from nucleation‐dominated to growth‐dominated processes due to the excess material availability. This occurs when existing particles preferentially capture more silver atoms instead of creating new nucleation sites.

Progressive coalescence was seen in thicker depositions (10–15 nm) (Figure 2d–f). At 10 nm, AgNPs reached 45 ± 7 nm (57% increase), while 15 nm showed the largest sizes (57 ± 13 nm) with a 70% reduction in particle density compared to the 5 nm. The systematic progression from 3 nm to 15 nm thickness reveals morphological evolution from optimally controlled nucleation (5 nm), through transitional growth‐dominated regimes (7–10 nm), to eventual loss of size control and reduced particle density at excessive thicknesses (12–15 nm). This transition from island growth to individual particle formation represents a thickness‐dependent nucleation phenomenon. While the specific threshold thickness may depend on deposition parameters, substrate surface characteristics, PVD equipment characteristics, and kinetic factors during ultra‐thin film growth, our results demonstrate that uniform NP coverage requires sufficient initial material to overcome the nucleation barriers presented by the hydrophobic surface.

This progression demonstrates the critical importance of precise thickness control in maintaining the delicate balance between sufficient material availability and controlled NP formation. For comparison, control substrates without fluorosilane treatment showed a significantly different morphology with irregular silver film formation rather than discrete NPs (Figure 2g; Figure S4f).

**FIGURE 3:**
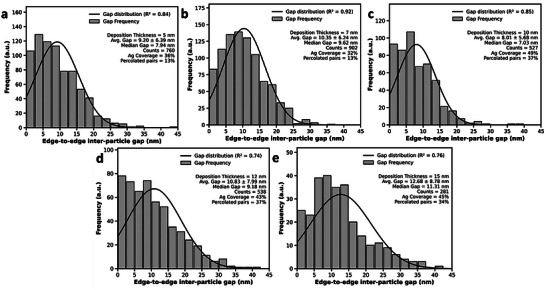
Edge‐to‐edge inter‐particle gap distributions extracted from SEM images of SILVERED substrates for silver deposition thicknesses of (a) 5 nm, (b) 7 nm, (c) 10 nm, (d) 12 nm, and (e) 15 nm. Histograms (gray bars) show the distribution of nearest‐neighbor gaps between well‐resolved particle pairs, with Gaussian fits (solid line; R^2^ as indicated). Inset values report mean ± SD, median, number of pairs analyzed (Counts), silver areal coverage (Ag Coverage), and the fraction of nearest‐neighbor pairs that are physically merged (Percolated pairs). Data are reported as mean ± SD; the number of well‐resolved particle pairs per thickness is given by the Counts value in each panel inset. Gaussian fits are reported with the R^2^.

To quantify how film morphology evolves with deposition thickness beyond particle size alone, automated edge‐to‐edge nearest‐neighbor analysis of the SEM images was performed for each thickness (Figure 3 and Table 1; Figure S1). Three metrics were extracted: the median inter‐particle gap, the fraction of nearest‐neighbor pairs separated by less than 10 nm (the regime in which coupled‐plasmon hot‐spot enhancement is maximized), and the fraction of pairs that are physically merged (percolated) rather than separated by an air gap. These three metrics together describe what fraction of the substrate is in the SERS‐active geometry as opposed to either tooopen or over‐coalesced.

**TABLE 1:** SEM‐derived morphological parameters of SILVERED substrates as a function of silver deposition thickness.

Deposition thickness (nm)	Avg. particle size (nm)	Avg. gap (nm)	Median gap (nm)	Gap < 10 nm (%)	Ag coverage (%)	Percolated pairs (%)
5	29.21 ± 4	9.20 ± 6	7.94	61	36	13
7	29.76 ± 6	10.35 ± 6	9.62	52	32	13
10	45.92 ± 7	8.01 ± 5	7.03	67	49	37
12	49.59 ± 7	10.83 ± 8	9.18	54	43	37
15	57.42 ± 13	12.68 ± 9	11.31	45	45	34

At 5 nm, the substrate occupies a distinct point in this morphological space: the median gap is 7.94 nm, 61% of all well‐resolved nearest‐neighbor pairs lie below 10 nm, and only 13% of pairs are percolated. This combination of small particles, narrow gaps, high gap density, and minimal percolation is unique among the thicknesses studied; no other deposition simultaneously satisfies all four conditions. At 7 nm, the median gap widens to 9.6 nm, and the sub‐10 nm pair fraction drops to 52%, reflecting the polydispersity increase already noted from the size distributions and consistent with the 2.3‐fold SERS performance drop reported in Figure 5.

Between 7 and 10 nm, a sharp morphological transition occurs, so the silver areal coverage jumps from 32% to 49% and the percolated‐pair fraction nearly triples, from 13% to 37%. Visually, the 10 nm image shows worm‐like merged structures that span large regions of the substrate. Above 10 nm, the apparent narrow median gap (7.0 nm at 10 nm) is therefore misleading; it counts only the surviving discrete pairs and is dominated by particles squeezed into the shrinking unconnected pockets, while more than a third of all nearest‐neighbor relationships are physically merged. From a SERS perspective, the merged regions support delocalized metallic‐film modes rather than localized gap‐plasmon hot spots, which is the physical origin of the enhancement collapse at 10–15 nm.

At 12 and 15 nm, surface‐energy‐driven dewetting begins to redistribute the silver: the particle count per unit analysis area falls from 760 at 5 nm to 538 at 12 nm and 281 at 15 nm, while the median gap widens to 9.2 nm and then 11.3 nm. Coverage stabilizes around 43%–45%, but the morphology is now a coarsened large‐island array in which neither small particles, narrow gaps, nor high gap density survive. Taken together, these morphological measurements rationalize the experimental focus of the SERS analysis on the 5 and 7 nm conditions; among the thicknesses studied, only the 5 and 7 nm substrates preserve the discrete‐particle architecture with a meaningful gap population required for coupled‐plasmon hot‐spot enhancement, with the 5 nm condition uniquely combining narrow gaps, high gap density, and minimal percolation. The 10, 12, and 15 nm substrates were therefore not pursued for analytical SERS, as their morphology, dominated by percolation at 10 nm and by coarsened large islands at 12–15 nm, places them outside the regime in which discrete gap‐plasmon hot spots can form.

The morphological optimum at 5 nm is consistent with the underlying plasmonic physics of AgNP arrays. The localized surface plasmon resonance (LSPR) of isolated spherical AgNPs in air is known to peak near 380–420 nm and to redshift with increasing particle size and dielectric environment [30], so the 29 nm particles obtained at both the 5 and 7 nm depositions are intrinsically detuned from 532 nm excitation. In dense AgNP arrays, however, the dominant enhancement is not driven by isolated‐particle resonance but by coupled‐plasmon modes localized in the inter‐particle gaps, where the near‐field intensity is known to scale steeply with decreasing edge‐to‐edge separation, and the gap‐plasmon resonance is shifted into the visible–near‐infrared regardless of single‐particle size [31]. The gap distribution and percolation statistics presented above (Table 1; Figure 3) place the 5 nm substrate squarely in this gap‐coupling regime, with the 7 nm condition retaining a weaker but still operative version of the same mechanism.

### 2.2 3D Architecture and Elemental Composition

**FIGURE 4:**
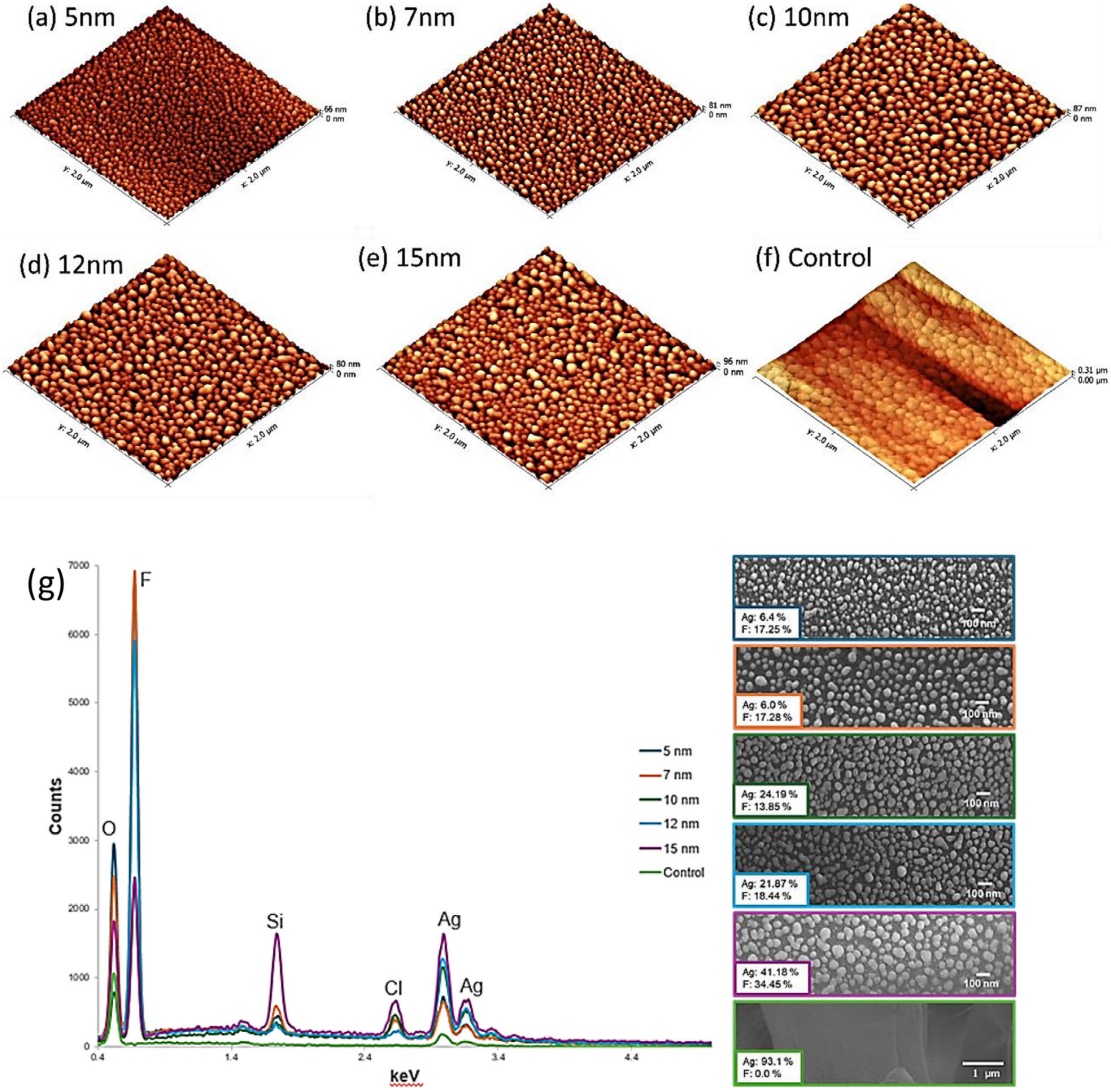
AFM topographical analysis showing 3D NP architecture. (a) 5 nm (66 nm height, 13‐fold amplification), (b) 7 nm (81 nm height), (c) 10 nm (87 nm height), (d) 15 nm (96 nm height). Scale bars: 500 nm. (g) BEX elemental analysis showing silver, fluorine, and silicon content across deposition thicknesses. F/Ag ratios demonstrate preserved surface chemistry despite increasing metal loading.

AFM topographical analysis revealed significant vertical amplification; 5 nm deposited silver transforms into 66 nm height particles (Figure 4a–f). This 13‐fold amplification has important implications for SERS performance. Vertically extended particles create deep electromagnetic field gradients extending into the solution phase, increasing the effective sensing volume by over an order of magnitude compared to planar film geometries. The vertical architecture generates “lightning rod” effects at particle tips [32], producing intense field localization sufficient for single‐molecule detection. The 10–20 nm distance between adjacent NPs forms optical nanocavities [33] where incident electromagnetic radiation becomes trapped and recirculates, further amplifying local field enhancement.

Elemental analysis showed silver content progression from 8.1% (5 nm) to 31.1% (15 nm) (Figure 4g). Despite only a 43% increase in deposited material, silver content increased 2.5‐fold between 7 and 10 nm, suggesting a regime in which incoming atoms are efficiently captured by sufficient particle density. Fluorine signals remained constant (11.2%–11.5%), indicating surface modification stability despite energetic atom exposure. BEX elemental mapping confirmed uniform distribution of silver, fluorine, and silicon across all deposition thicknesses, with no evidence of elemental segregation or localized depletion (Figure S2).

The ratios of fluorine to silver reveal that at 5 nm (F/Ag = 1.38), fluorine dominates while maintaining hydrophobicity; at 15 nm (F/Ag = 0.37), silver dominates but maintains hydrophobicity (CA > 110°). It should be noted that BEX quantification on topographically complex surfaces is subject to artifacts from variable interaction volumes and x‐ray shadowing/reabsorption by surface features, particularly at higher NP densities. Therefore, these values represent semi‐quantitative trends rather than absolute surface composition. Nonetheless, this architectural configuration creates dual‐function surfaces, with fluorinated areas between particles maintaining their hydrophobic nature for analyte concentration and silver islands offering plasmonic enhancement. In fact, the resulting topography essentially engineers surfaces where “valleys” repel aqueous solutions and “peaks” enhance Raman signals, creating synergistic effects that amplify sensitivity beyond individual component contributions.

### 2.3 SERS Performance and Analytical Metrics

**FIGURE 5:**
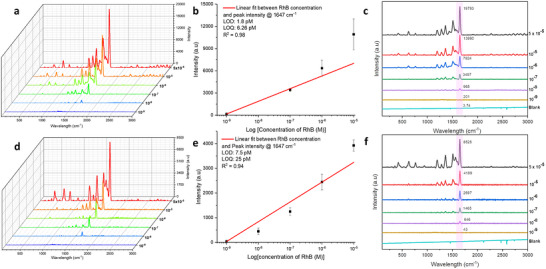
SERS performance for RhB detection. (a and d) Concentration‐dependent Raman spectra spanning 10^−9^ m to 5 × 10^−5^ m for 5 nm and 7 nm substrates, respectively. (b and e) Calibration curves showing linear response: 5 nm substrate (R^2^ = 0.98) versus 7 nm substrate (R^2^ = 0.94), respectively. (c and f) Comparative signal intensity analysis at 1647 cm^−1^ for both thicknesses.

**FIGURE 6:**
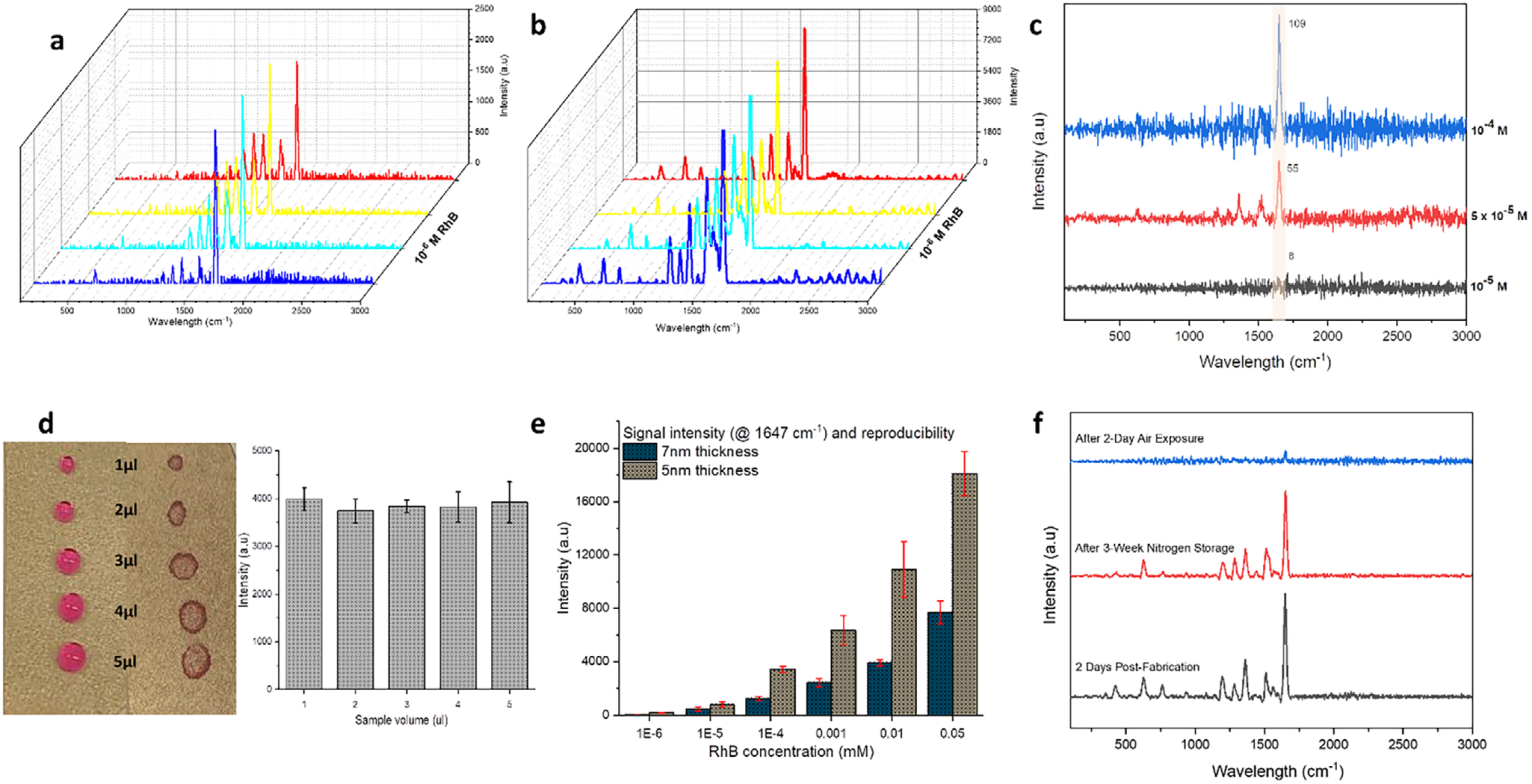
Reproducibility and stability assessment. (a, b) Spot‐to‐spot variation across 20 randomly selected locations for 7 nm and 5 nm thicknesses, respectively, showing RSD <10%. (c) SERS spectra from 7 nm silver‐coated hydrophilic control substrate. (d) Volume‐independent performance across 1–5 µL sample volumes. (e) Comparative signal intensity analysis for 5 nm and 7 nm thicknesses. (f) Temporal stability: freshly prepared substrate, 3‐week nitrogen storage (>90% signal retention), and 2‐day ambient air exposure (complete signal loss).

The reproducibility and analytical performance reported below were assessed on at least three independently fabricated SILVERED substrates per deposition thickness, produced on separate days, so that the reported linearity, spot‐to‐spot variability, and dose‐response statistics implicitly incorporate batch‐to‐batch contributions to variance. Performance evaluation using RhB demonstrated strong sensitivity. The 5 nm substrate showed peak intensities from 201 at 10^−9^ m to 19 793 at 5 × 10^−5^ m, maintaining linearity across four orders of magnitude (R^2^ = 0.98) (Figure 5a–c). This linear dynamic range spanning four decades of concentration is essential for practical analytical applications, where environmental or biological samples may contain target analytes at concentrations varying from 10^−12^ to 10^−6^ m. Through controlled deposition, the 2 pm detection limit provides superior reproducibility while surpassing many chemically synthesized substrates (Table 2). The EF of 10^6^ demonstrates strong enhancement suitable for ultrasensitive detection. The control substrate used for the AEF calculation is a silver‐deposited (7 nm), untreated, hydrophilic filter paper (Figure 6c; Figure S3), chosen to isolate the contribution of the fluorosilane‐driven AgNP morphology rather than the trivial contribution of having silver present at all. Using a silver‐free paper control would conflate the plasmonic enhancement attributable to the SILVERED architecture with the absence of any plasmonic material and would overestimate the SILVERED contribution accordingly. This enhancement corresponds to sensitivity improvements from µm to pm concentration ranges. For clinical diagnostics, this sensitivity range encompasses disease biomarker concentrations relevant for early‐stage disease detection [10].

**TABLE 2:** Comparative analysis of SERS substrates for RhB detection.

Platform	LOD	EF	Fabrication method	Stability	Cost/complexity	Refs.
Al + NiAl + Ag flexible	10^−14^ M	2.05 × 10^17^	Multi‐step deposition	Not reported	High	[40]
Hollow Au NPs	10^−^^1^ ^8^ M	10^11^	Complex synthesis	1 year	Very high	[41]
Ag‐decorated Si photonic	10^−10^ M	10^10^	Multi‐layer deposition	Not reported	High	[42]
N_2_‐doped reduced GO	10^−6^ M	10^3^	Wet chemical synthesis	Not reported	low	[43]
Ag hierarchical structures	10^−8^ M	Not reported	Wet chemical synthesis	50 days	Moderate	[44]
SILVERED	10^−12^ M	10^6^	Single‐step deposition	Stable under nitrogen	Very low	This work

The 7 nm substrate exhibited a 2.3‐fold decrease in performance, with the peak intensity range of 43–8525 (Figure 5d–f). The reduction to 10^5^ enhancements despite only 2 nm additional deposition demonstrates the extreme sensitivity of SERS performance to morphological parameters. This degradation results from particle coalescence reducing the density of sub‐10 nm hot spots between adjacent AgNP where most electromagnetic enhancement occurs.

RSDs were consistently below 10% at 20 locations (average 7.5%) according to reproducibility assessment (Figure 6a,b,e). Volume independence (1–5 µL) showed consistent intensities (3900 ± 200) (Figure 6d). The elimination of coffee‐ring effects through hydrophobic surface engineering solves a persistent challenge with low spectral reproducibility in droplet‐based SERS measurements [34]. The maintained high CA throughout evaporation causes uniform droplet shrinkage toward the center rather than outward capillary flow. This “self‐focusing” behavior ensures that analyte molecules converge on single measurement locations with uniform spatial distribution, eliminating the need for precise volume control or systematic searching for optimal measurement positions. The intense magenta color of RhB serves as a direct visual reporter of analyte distribution; under identical drop‐cast conditions (5 µL of the same RhB solution), the deposited dye wicks laterally through the cellulose fibers and disperses across a large diffuse area on the untreated hydrophilic control substrate (Figure S3), whereas on the fluorosilanized SILVERED substrate (Figure S4) it remains pinned as a compact, sharply bounded magenta spot with no lateral spreading. This direct visual comparison, in which the only experimental variable is the fluorosilane treatment, provides macroscopic evidence of the self‐focusing behavior, independently validated by the volume‐independent SERS response across 1–5 µL droplets (Figure 6d) and the low spot‐to‐spot RSD measured within the deposit (Figure 6a,b). The use of uniform hydrophobicity for analyte focusing distinguishes SILVERED from earlier patterned‐wettability paper‐SERS designs such as the PLLA/gold‐nanorod substrate of Shao et al. [35]. In which a discrete hydrophilic patch is required to pin the droplet onto the SERS‐active area. In SILVERED, the entire substrate is uniformly hydrophobic, and the focusing effect emerges from droplet evaporation dynamics rather than from a pre‐defined wettability pattern, eliminating the need for patterning steps and enabling direct scaling to commercial cellulose paper substrates.

Temporal stability revealed distinct requirements: more than 95% retention after almost one month under nitrogen, but complete loss after 2 days of ambient exposure (Figure 6f). The rapid ambient degradation results from the high surface‐to‐volume ratio of 29 nm NPs, which makes them highly susceptible to oxidation. Atmospheric oxygen forms nanometer‐scale Ag_2_O surface layers that completely decouple the plasmonic resonance by converting metallic silver to its oxide, effectively “switching off” the optical nanoantenna function [31, 36]. Atmospheric CO_2_ contributes a second pathway by forming Ag_2_CO_3_ at the silver surface, identified as a significant carbon‐contamination component in XPS studies of air‐exposed AgNP SERS substrates [36]. Trace atmospheric sulfur compounds, particularly hydrogen sulfide (H_2_S), have been shown to further accelerate this process by forming Ag_2_S surface layers that contribute to LSPR damping in parallel with oxidation [37]. The oxidation rate is mediated by water adsorbed at the silver surface, which explains the practical observation that nitrogen‐purged dry storage essentially halts degradation while ambient laboratory humidity completes the loss within 2 days. This points directly to vacuum‐ or inert‐gas‐sealed packaging, for example, hermetically sealed chips or blister‐packed units widely used in lateral‐flow and point‐of‐care assays, and to thin protective coatings such as Al_2_O_3_ atomic‐layer deposition [38], alkanethiol self‐assembled monolayers, or silica shells [39] as the natural mitigation strategies. Systematic optimization of these coatings is the subject of a dedicated follow‐up study, since each coating chemistry produces its own environmental response curve and would substantially expand the scope of the present work.

The single‐step PVD fabrication offers reproducibility advantages over wet chemical synthesis. Although Table 2 shows that higher enhancement factors have been reported for some solution‐deposited nanostar arrays and hybrid plasmonic–dielectric architectures, these typically come at the cost of multi‐step colloidal synthesis, sub‐wafer‐scale fabrication, or specialized substrates incompatible with cellulose‐based materials.

The 10^6^ AEF by SILVERED is achieved through straightforward modification without complex assembly and is delivered through a single dry‐process step that scales to large wafers at low per‐test cost, prioritizing manufacturability over peak enhancement. The combination of low fabrication cost, single‐step processing, and pM sensitivity positions SILVERED as accessible for laboratories without specialized nanomaterial synthesis capabilities, addressing the needs of resource‐limited settings while maintaining analytical performance suitable for biomedical detection, environmental monitoring and food safety applications.

### 2.4 Beyond SERS: Platform Versatility and Future Directions

While this study focused on SERS optimization, the unique properties of SILVERED substrates, controlled 29 nm AgNP morphology (PDI = 0.13), high particle density, and retained hydrophobicity, suggest potential for expanded applications. The uniform plasmonic properties may enable colorimetric sensing through LSPR shifts upon analyte binding [45, 46]. Silver's inherent antimicrobial properties combined with high surface area and hydrophobic modification warrant investigation for antibacterial applications in wound healing or antibiofilm applications [47, 48]. The catalytic activity of AgNPs for reactions suggests potential applications, facilitated by the paper's porous structure enabling reactant transport [49]. Future work could also explore multifunctional integration, combining detection with catalytic degradation for environmental sensors, biomedical sensing, or merging antimicrobial properties with colorimetric/SERS indicators for smart medical devices. Critical challenges requiring investigation include improving ambient stability beyond current limitations through protective coatings or self‐assembled monolayers, and systematically determining optimal fabrication parameters for each application domain.

## 3 Conclusions

This study demonstrates that the SILVERED method provides a robust, scalable approach for fabricating high‐performance SERS substrates through precise control of surface chemistry and deposition parameters. The optimal 5 nm silver deposition on fluorosilanized paper achieves uniform NP morphology (PDI = 0.13), high particle density, and exceptional analytical performance (LOD = 2 pm, EF = 10^6^). Quantitative SEM image analysis confirms that the 5 nm condition uniquely combines narrow inter‐particle gaps, high gap density, and minimal percolation, establishing a direct structure–performance link between film morphology and the SERS optimum. The reported analytical metrics were established across at least three independently fabricated batches of 5 and 7 nm substrates and remained reproducible over nearly one month under nitrogen storage, supporting the platform's practical reliability. Our systematic investigation reveals that even minor deviations from optimal thickness result in significant performance degradation, emphasizing the importance of precise parameter control. The SILVERED method's simplicity, scalability (up to 12‐inch substrates), and elimination of post‐processing steps with low production cost position it as a versatile platform technology. The fundamental insights into surface energy‐controlled NP formation provide design principles applicable to diverse nanomaterial systems and establish a framework for rational design of plasmonic substrates through thermodynamic control of nucleation and growth processes. Beyond SERS applications, the controlled NP morphology and hydrophobic surface present opportunities for colorimetric sensing, catalytic applications, antimicrobial surfaces, and microfluidic integration.

## 4 Materials and Methods

### 4.1 Materials

Perfluorooctyltrichlorosilane (FS, 97%) was purchased from Thermo Fisher. Whatman No. 5 filter paper and RhB were obtained from Millipore Sigma. All materials were used without further purification.

### 4.2 SILVERED Substrate Fabrication

Filter paper substrates were treated with fluorosilane through drop casting in a batch chamber for 24 h at room temperature to create hydrophobic surfaces. Silver was deposited using physical vapor deposition (Lesker PVD75 E‐Beam/Thermal Evaporator) at nominal thicknesses (3, 5, 7, 10, 12, 15 nm) under high vacuum (≈10^−8 ^Torr). Deposition rate was maintained at 14 mA, 0.1 Å/s, and monitored using quartz crystal microbalance. At least three independently fabricated batches of substrates were produced on separate days for each of the deposition thicknesses studied.

### 4.3 SERS Measurements

For SERS analysis, RhB solutions at various concentrations were applied to the SILVERED substrates with 5 nm and 7 nm thicknesses and allowed to dry for 2 h at room temperature before measurement (Figures S4 and S5). Raman spectra were acquired using a WiTec confocal Raman microscope equipped with a 532 nm laser excitation source. Measurements were performed at 0.05 mW laser power to prevent sample damage while maintaining sufficient signal intensity. The SERS EF of the nanoprobes was assessed by calculating the analytical EF (AEF) using the following equation [10]:

(1) $$AEF=\frac{C_{Raman}I_{SERS\;}}{I_{Raman}C_{SERS}}$$

where *I_SERS_* and *I_Raman_* represent the peak intensities obtained from Ag‐deposited treated substrate and Ag‐deposited control substrate, respectively, while *C_SERS_* and *C_Raman_* denote the lowest detectable RhB concentrations in SERS substrate and control substrate, respectively. For the I_Raman_ /C_Raman_ measurement, RhB was applied to the Ag‐deposited control substrate at 5 × 10^−5^ m, the lowest concentration at which a measurable Raman signal could be obtained on the control; this concentration was used together with the lowest detectable RhB concentration on the SILVERED substrate (2 pm for the 5 nm condition) to calculate the AEF. For each substrate, SERS spectra were collected at *n* = 20 randomly selected locations to assess spot‐to‐spot variability (reported as RSD in section 2.3), and substrate‐to‐substrate variability is implicitly captured in the reported metrics because each data set was acquired across at least three independently fabricated 5 and 7 nm substrates. Aging effects were assessed by acquiring SERS spectra on substrates stored for up to nearly one month under nitrogen and on substrates exposed to ambient laboratory air, as reported in Figure 6f. All SERS measurements and contact‐angle measurements were performed under ambient laboratory conditions at 22 ± 2°C and 30%–50% relative humidity. Substrate drying after analyte drop‐casting was carried out under a nitrogen atmosphere (1 h) to prevent atmospheric oxidation during the drying step. Between fabrication and measurement, substrates were stored in sealed containers under nitrogen at room temperature.

### 4.4 Substrate Characterization

Scanning electron microscopy (SEM) and elemental analysis were performed with a JEOL JSM‐IT800HL FESEM and Oxford Instruments Unity BEX hybrid BSE/x‐ray detector. High‐resolution SEM images and x‐ray spectra were acquired at 10 keV with a 10 mm working distance. AC mode Atomic Force Microscopy (AFM) was performed with an Oxford Instruments Asylum MFP‐3D. Nominal cantilever and tip parameters were 300 kHz, 40 N/m, and ∼10 nm diameter. Image analysis was performed using ImageJ (SEM) and Gwyddion (AFM) software, and graphs were plotted with OriginPro 9.5.

### 4.5 Image Analysis

Inter‐particle gap statistics and silver areal coverage were extracted from SEM images (Figure S1) using a custom Python (v3.11) pipeline based on scikit‐image (v0.26), SciPy (v1.13), and NumPy (v1.26). The pixel‐to‐nanometer scale was calibrated automatically from the embedded scale bar for each image. After Gaussian smoothing (σ = 1 pixel), images were binarized using the Otsu threshold (with Li thresholding computed in parallel as a robustness check), and small artifacts (connected components <15 pixels; internal holes <10 pixels) were removed. Individual particles were segmented by marker‐controlled watershed on the Euclidean distance transform, with the seed separation tuned per image so that the median equivalent‐area diameter matched the reference particle size (29 nm at 5 nm deposition); features with equivalent diameters outside 8–80 nm were rejected. To eliminate boundary bias, particles whose centroids lay within a buffer of (mean diameter + 2 SD) of any image edge were excluded as query particles, though they remained available as neighbor candidates. For each interior particle, the nearest neighbor was identified using a k‐d tree (scipy.spatial.cKDTree), and the edge‐to‐edge gap was computed as the centroid‐to‐centroid distance minus the sum of the two area‐equivalent radii. Pairs with non‐negative gap values were classified as well‐resolved, while pairs with negative values were classified as percolated (physically merged or below segmentation resolution) and reported separately. Silver areal coverage was computed as the ratio of bright pixels to total pixels in the cleaned binary mask.

### 4.6 Statistical Analysis

Quantitative data are reported as mean ± SD, with sample sizes (n) stated in the corresponding figure legends and table footnotes. Raman pre‐processing was limited to baseline subtraction; no normalization, transformation, or outlier removal was applied. The LOD was calculated as 3σ/slope, where σ is the SD of *n* = 4 blank measurements, and the slope is the linear regression coefficient of the calibration curve; calibration curves are reported with the R^2^, slope, and 95% confidence interval. Spot‐to‐spot variability is expressed as the relative standard deviation (RSD = SD/mean × 100%). For the SEM image analysis, the combined uncertainty on the mean inter‐particle gap at each deposition thickness was estimated as the quadrature sum of the statistical uncertainty (SD divided by the square root of the well‐resolved pair count) and the threshold‐systematic uncertainty (the absolute difference between mean gaps computed with Otsu and Li thresholding). The present study reports descriptive statistics (calibration regression, RSD, and morphological distributions) rather than between‐group hypothesis tests; no significance testing, post‐hoc adjustment, or *p* values are reported. Statistical analysis was performed using OriginPro 9.5 and Python 3.11 with SciPy 1.13.

### 4.7 Generative AI Disclosure

Generative artificial intelligence tools (ChatGPT, OpenAI GPT‐4) were utilized to refine the language and correct grammatical errors in this manuscript. The use of AI was limited to improving writing quality and clarity; all scientific content, experimental design, data collection, analysis, and interpretation were performed exclusively by the authors.

## Author Contributions

**Farbod Ebrahimi**: conceptualization, methodology, investigation, image analysis, data curation, writing – original draft, review, and editing. **Anjali Kumari**: data curation and analysis, photo design, review, and editing. **Kyle Nowlin**: data curation and analysis, review, and editing. **Tohid Didar**: supervision, review, editing. **Kristen Dellinger**: conceptualization, funding acquisition, supervision, writing, review, and editing. All authors have approved the final version of the manuscript.

## Conflicts of Interest

The authors declare no conflicts of interest.

## Supporting information




**Supporting File**: smtd70803‐sup‐0001‐SuppMat.docx.

## Data Availability

The data that support the findings of this study are available from the corresponding author upon reasonable request.
