# Supplementary material for: Transforming Paper into Plasmonic Sensors: One‐Step Fabrication of High‐Enhancement SERS Nanosubstrates via Surface Energy Control
Source: Small Methods. 2026 Jun 26;10(14):e70803. doi: 10.1002/smtd.70803 (PMC13397137; doi:10.1002/smtd.70803)
Supplement: Supplementary file 1 — Supporting File: smtd70803‐sup‐0001‐SuppMat.docx. [file SMTD-10-e70803-s001.docx]

**Supplementary information**

Transforming Paper into Plasmonic Sensors: One-Step Fabrication of High-Enhancement SERS Substrates via Surface Energy Control

*Farbod Ebrahimi^1^, Anjali Kumari^1^, Kyle Nowlin^2^, Tohid Didar,^3,*^, Kristen Dellinger^1,*^*

^1^Department of Nanoengineering, Joint School of Nanoscience and Nanoengineering, North Carolina A&T State University, 2907 East Gate City Boulevard, Greensboro, NC, 27401 USA,

^2^Department of Nanoscience, Joint School of Nanoscience and Nanoengineering, University of North Carolina at Greensboro, 2907 East Gate City Boulevard, Greensboro, NC, 27401 USA

^3^Department of Mechanical Engineering, McMaster University, 1280 Main St W, Hamilton, Ontario, L8S 4L8 Canada

**Content:**

Figures S1 to S5

Supplementary method

**SEM for images analysis**

| **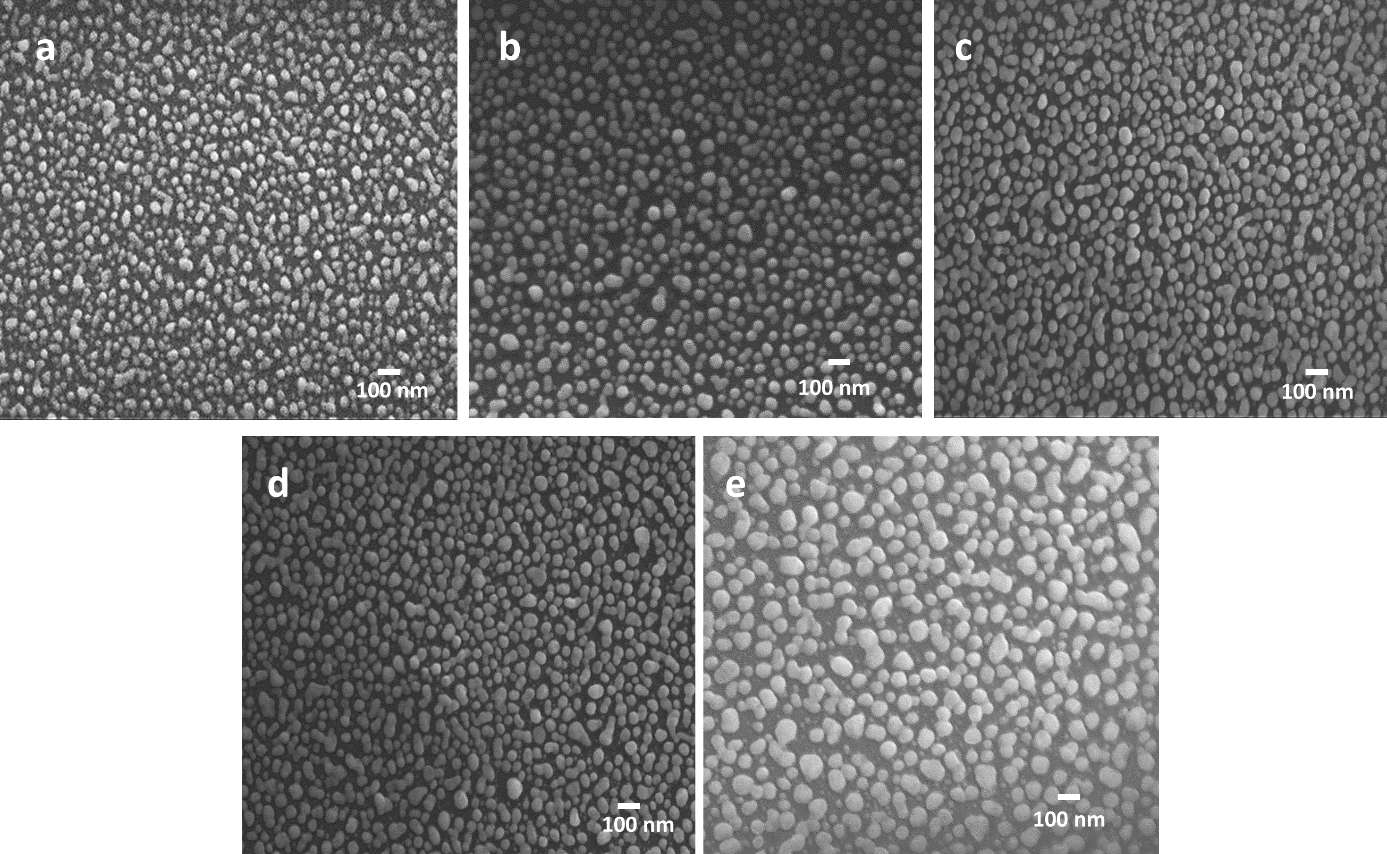** |
| --- |
| Figure S1. Representative SEM images of SILVERED substrates used for the inter-particle gap analysis, at silver deposition thicknesses of (a) 5 nm, (b) 7 nm, (c) 10 nm, (d) 12 nm, and (e) 15 nm. Scale bars: 100 nm. |

**BEX - Mapping**

| 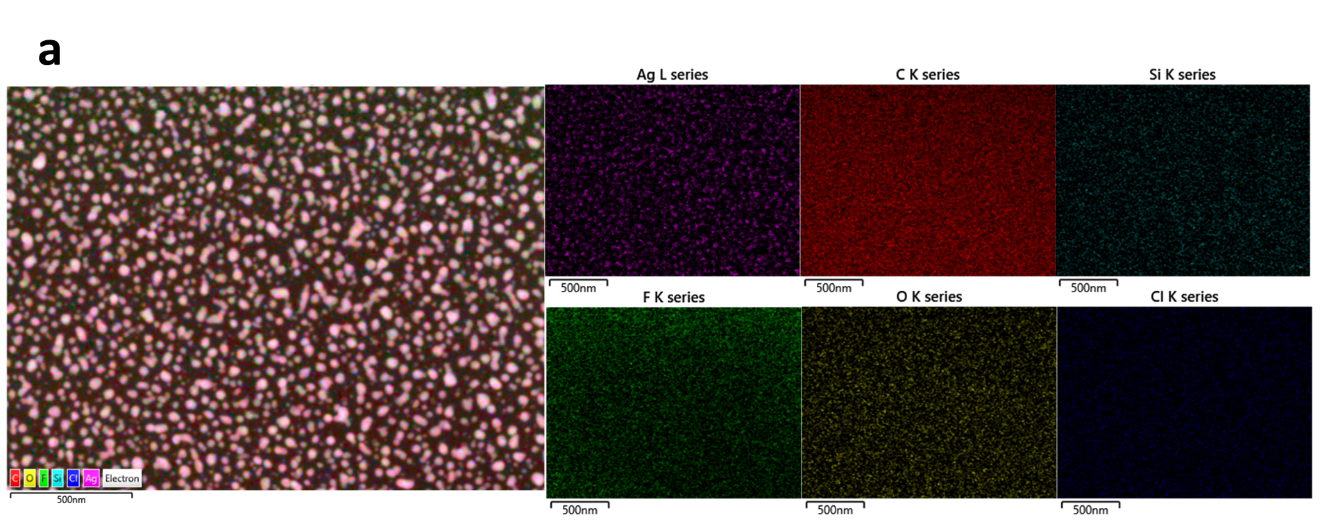 |
| --- |
| 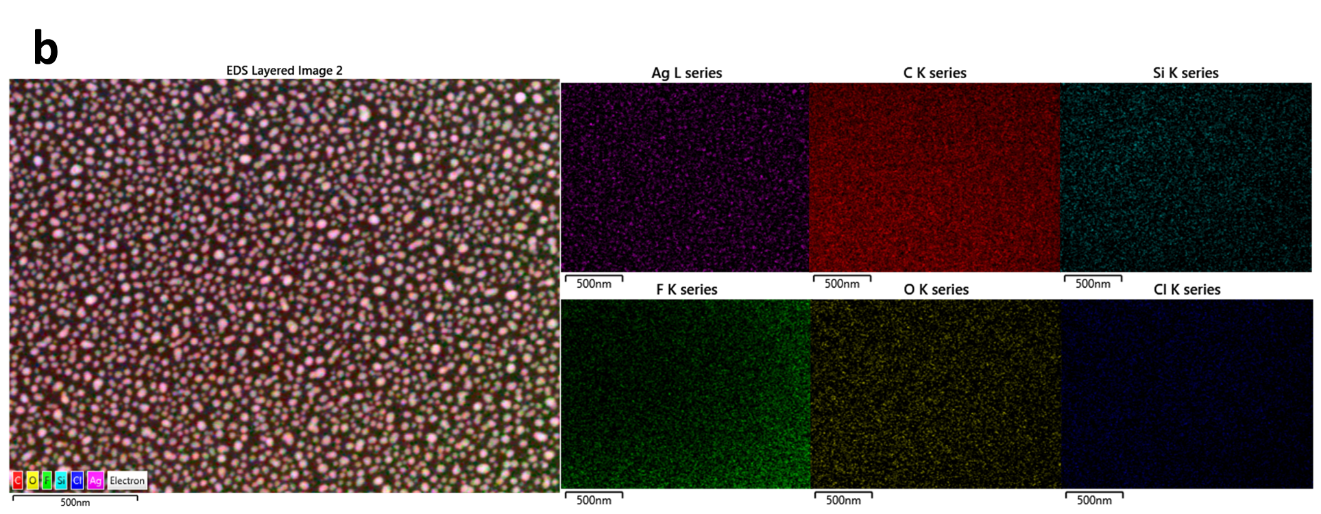 |
| 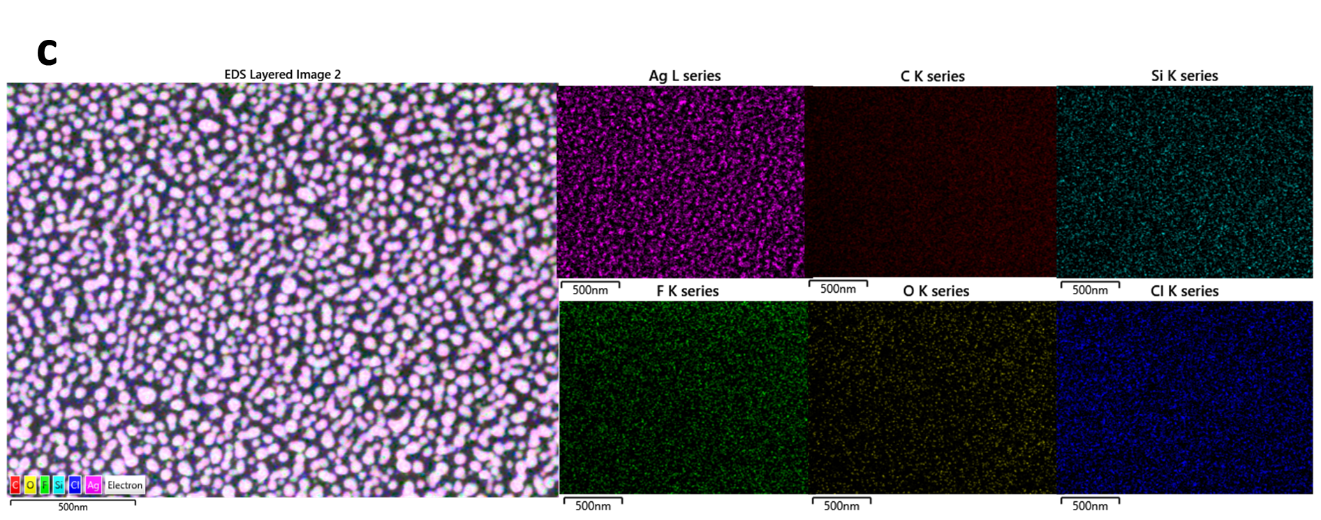 |
| 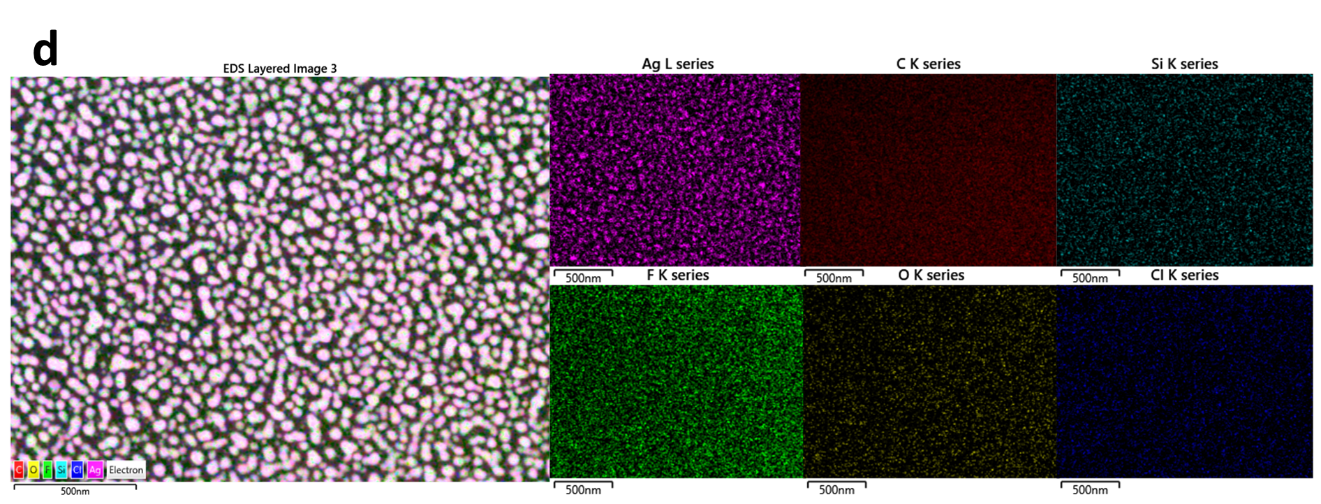 |
| 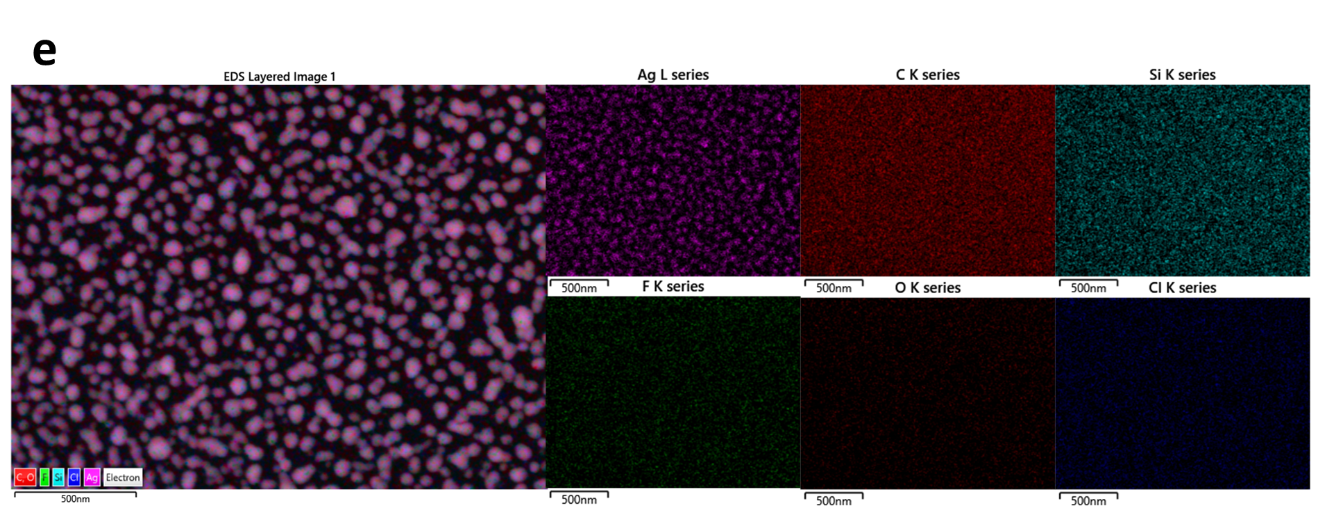 |
| 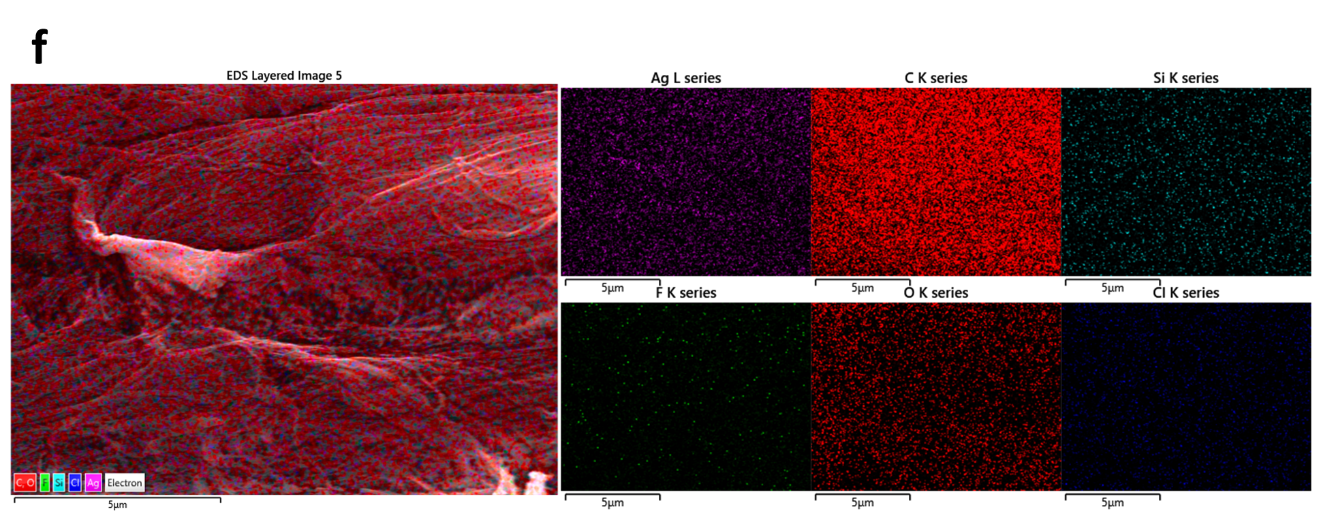 |
| Figure S2. BEX elemental mapping of SILVERED substrates at various silver deposition thicknesses. Panels show spatial distribution of silver (Ag), fluorine (F), silicon (Si), carbon (C), chlorine (Cl) and oxygen (O) across substrates with: (a) 5 nm, (b) 7 nm, (c) 10 nm, (d) 12 nm, (e) 15 nm nominal silver deposition thicknesses, and (f) control substrate without fluorosilane treatment (7 nm silver deposition on untreated paper). The uniform distribution of fluorine and silicon across fluorosilanized samples (a-e) confirms stable surface modification throughout the deposition process, while their absence in the control (f) validates the surface treatment specificity. |

**Supplementary Methods**

**Sample Preparation for SERS Measurements**

SILVERED substrates were cut into sections using clean scissors. RhB solutions at desired concentrations were prepared by serial dilution in DI water. For each measurement, 5 µL of analyte solution was carefully pipetted onto the substrate center using a micropipette, and substrates were placed in a covered Petri dish to prevent airborne contamination during drying. Samples were allowed to dry for 2 h at room temperature in nitrogen box before Raman measurement.

**Sample preparation – Control substrates**

| 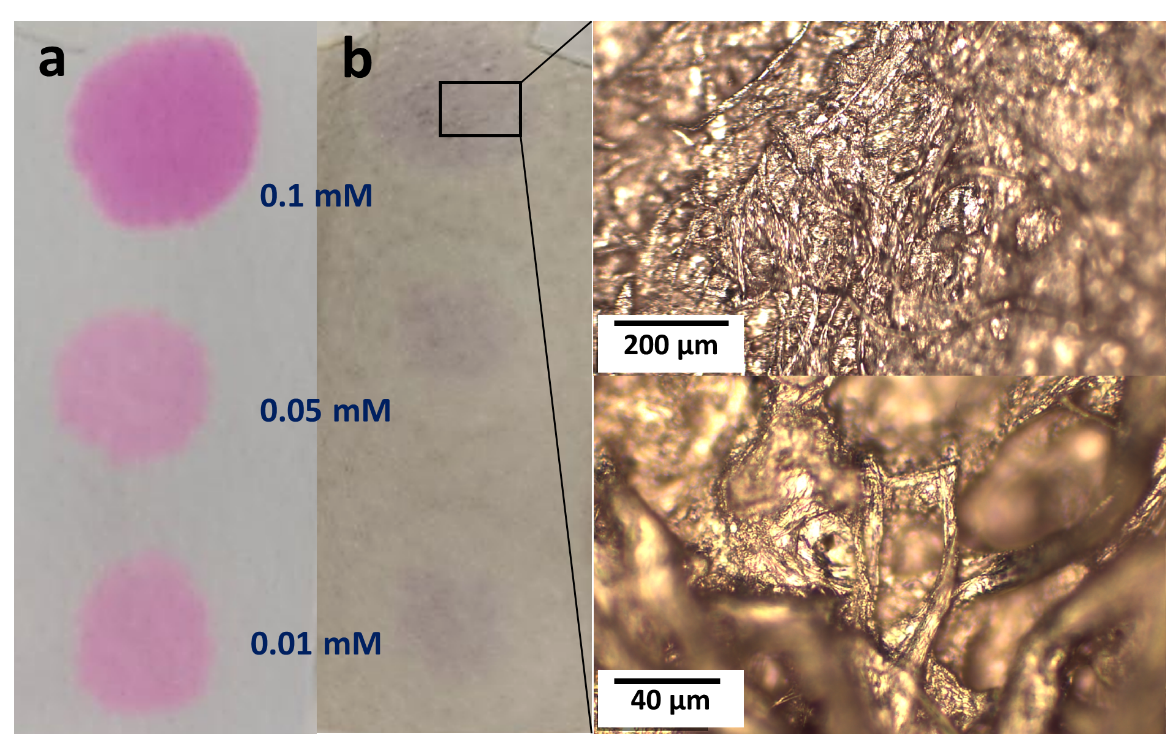 |
| --- |
| Figure S3. Control substrates, a) normal filter paper, b) untreated hydrophilic paper silver-coated with 7 nm thickness and Raman WiTech microscopy image. |

**Sample preparation – 5 nm substrates**

| 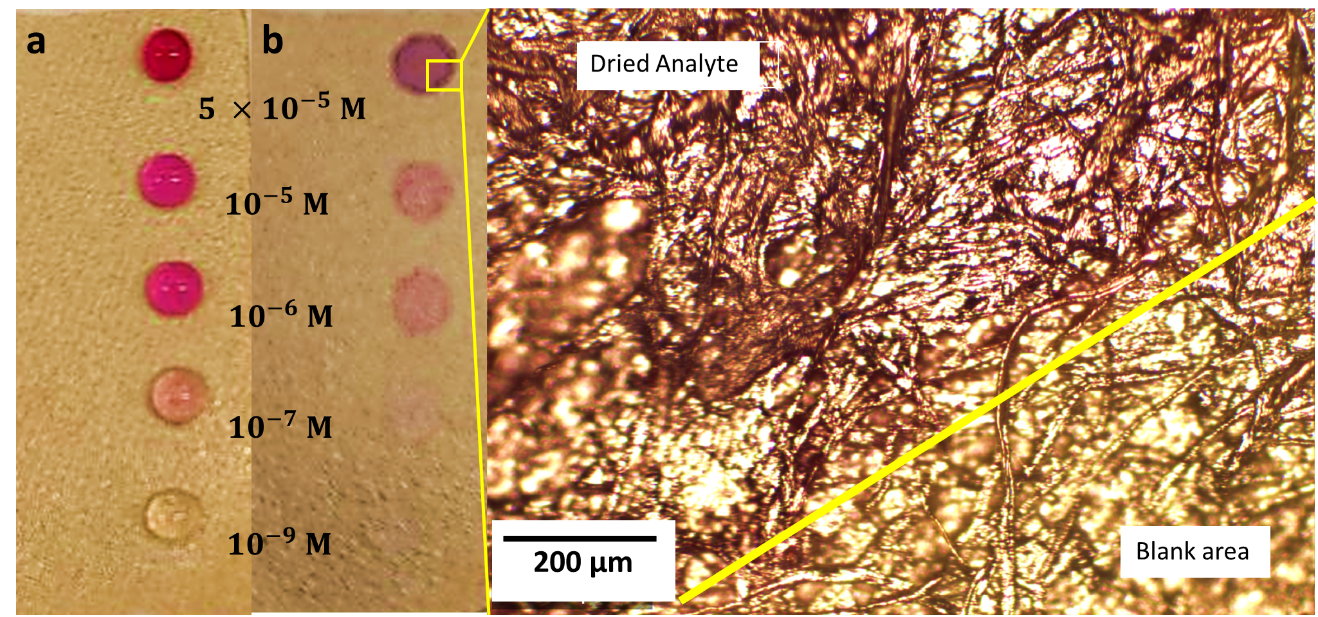 |
| --- |
| Figure S4. The 5 nm silver-coated hydrophobic Nanosubstrate , a) 5 µl of different concentration of RhB, b) dried sample after 2 h under nitrogen ambient and Raman WiTech microscopy from sample area and blank area. |

**Sample preparation – 7 nm substrates**

| 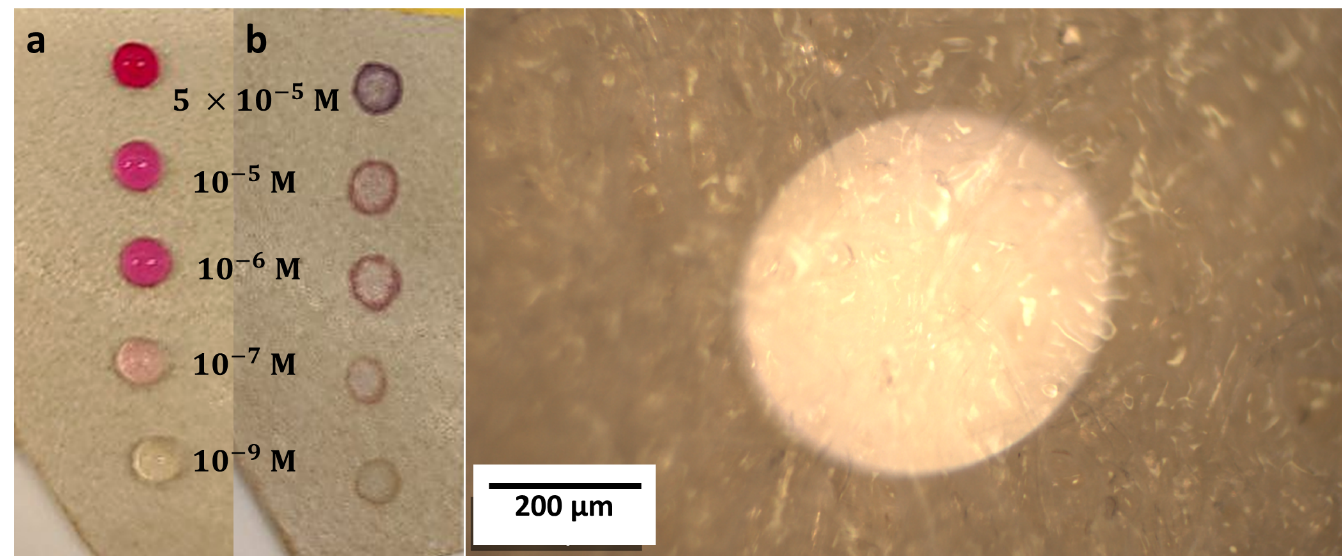 |
| --- |
| Figure S5. The 7 nm silver-coated hydrophobic Nanosubstrate , a) 5 µl of different concentration of RhB, b) dried sample after 2 h under nitrogen ambient and Raman WiTech microscopy from droplet. |
